# Intravascular organizing thrombus in the forearm: a unique imaging presentation

**DOI:** 10.1007/s00256-025-05044-8

**Published:** 2025-10-10

**Authors:** Rachel Bass, Priyanka Mitta, Constantine Burgan, John Huffman, Bonnie Moore, Thomas Winokur

**Affiliations:** 1 Department of Radiology, University of Alabama at Birmingham, 619 19th Street South, JTN 304, Birmingham, AL 35249 USA; 2 School of Medicine, University of Alabama at Birmingham, 1620 University Boulevard, Birmingham, AL 35233 USA; 3 Department of Radiology, University of Alabama at Birmingham, 619 19th Street South, JTN 338, Birmingham, AL 35249 USA; 4 Department of Pathology, University of Alabama at Birmingham, 619 19th Street South, NP 3541, Birmingham, AL 35249 USA

**Keywords:** Organizing thrombus, Intravascular papillary endothelial hyperplasia, Masson’s tumor, Intravascular tumor

## Abstract

Organizing thrombus is a well described histologic finding involving the reorganizing and recanalization of a healing thrombus. However, it is rarely large enough to be described on imaging. When the thrombus becomes sufficiently large, intravascular papillary endothelial hyperplasia (IPEH) must be considered. IPEH or Masson’s tumor is a benign, exuberant form of organizing thrombus that typically occurs in the soft tissues of the head, neck, and distal extremities. It can occur within a vessel, hematoma, or vascular mass and can mimic benign processes or malignant lesions such as angiosarcoma. Histopathological characteristics are needed for final diagnosis. The treatment is typically complete surgical resection with excellent prognosis. We report the case of a patient presenting with swelling in his left forearm in the same site as a previously treated left basilic vein thrombus. Ultrasound demonstrated a noncompressible, heterogeneous intravascular mass with internal vascularity. Subsequent MRI showed an enhancing mass centered in the left basilic vein with a flow void with the differential diagnosis of IPEH versus intravascular neoplasm. Percutaneous biopsy was performed with histology consistent with reactive vascular proliferation and organizing thrombus. The papillary architecture diagnostic of IPEH was absent. Regardless of final specific terminology, this case presents exuberant organizing thrombus as a benign cause of an enhancing intravascular mass. This patient was treated conservatively with no further intervention.

## Introduction

An organizing thrombus refers to the change that a thrombus undergoes over time as it forms structured, vascularized connective tissue and becomes recanalized [1]. Organizing thrombus is a common histologic finding occurring in the setting of vessel wall injury or hematoma, and is not well characterized by imaging [2]. Intravascular papillary endothelial hyperplasia (IPEH) is a benign intravascular proliferation, which is now recognized as an exuberant form of organizing thrombus and imaging characteristics have been described on MRI and ultrasound modalities [3–5]. Treatment for IPEH is essentially curative with simple excision [6].

IPEH is rare, composing about 2% of all vascular tumors. These masses typically occur in the superficial soft tissues of the head, neck, and distal extremities, but can occur in any part of the body [7]. IPEH classically presents clinically as a singular, slow-growing, asymptomatic or tender nodule that may have a bluish or reddish skin appearance [8]. The significance of IPEH is its histopathologic similarity to malignant vascular neoplasms, such as angiosarcoma or Kaposi sarcoma, which can be distinguished by a trained pathologist. We present a case of organizing thrombus suspected to represent IPEH with an atypical histopathological appearance.

## Case report

A 61-year-old man with a past medical history of Miller Fisher syndrome managed with IVIG therapy presented with a 2-week history of swelling in his left forearm. Physical examination revealed an approximately 1 X 2 cm soft, nontender swelling of the medial left forearm with no discoloration. Eleven months prior to presentation, he developed sudden onset pain and cordlike swelling in the same area of his left forearm and was diagnosed with occlusive superficial thrombosis of the left basilic vein. This was treated with 2 months of Xarelto with resolution of symptoms.

**Fig. 1:**
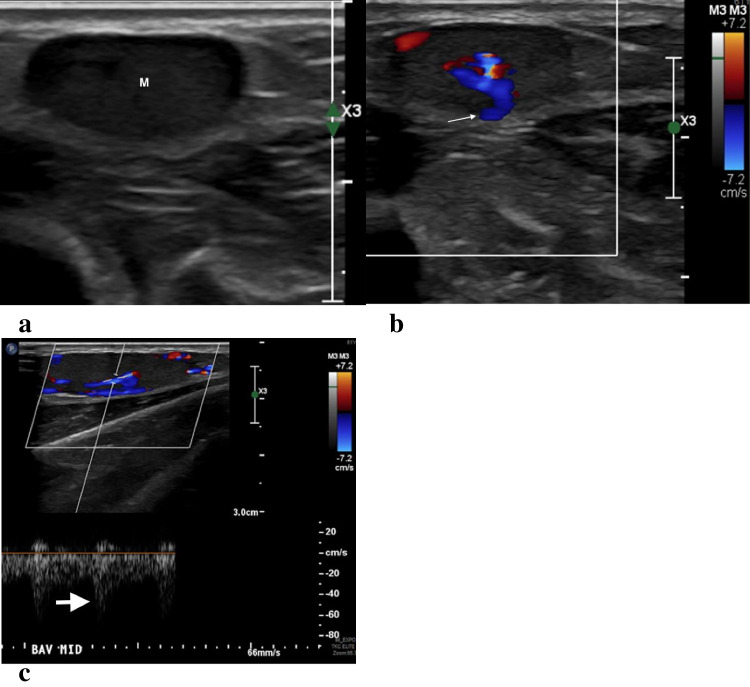
Recurrent intravascular mass in a 61-year-old man with previously treated venous thrombus. Grayscale (**a**), color Doppler (**b**) and spectral Doppler (**c**) images through the basilic vein demonstrate a fusiform hypoechoic mass (M) filling the vein with internal flow (thin arrow). The waveform is arterialized (thick arrow), which is atypical for a venous structure

**Fig. 2:**
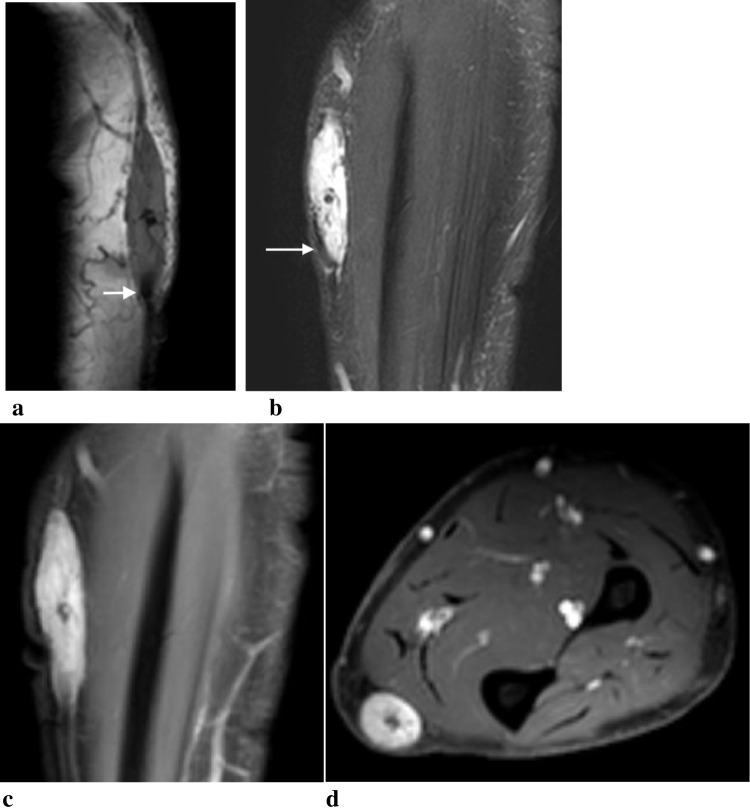
Fusiform intravascular mass filling the basilic vein corresponds to the palpable abnormality and follows signal characteristics of a vascular mass with a flow void (arrows) on sagittal T1 (**a**), coronal T2 fat saturated sequences (**b**). There is near diffuse internal enhancement on coronal and axial fat suppressed post contrast T1 sequences with central nonenhancement, representing residual thrombus or fibrous core (**c**, **d**)

On ultrasound, a vascular mass was confirmed in the basilic vein in the same location as prior thrombus (Fig. 1). Further imaging was obtained with magnetic resonance imaging (MRI) with and without contrast which showed an enhancing subcutaneous mass centered on the basilic vein without surrounding edema. The mass was T1 isointense and diffusely hyperintense on T2 fat saturated sequences with several hypointense internal septations. There was homogenous enhancement on post-contrast T1and focal hypointense flow void seen on all sequences (Fig. 2). Based on imaging findings, differential considerations included IPEH or intravascular neoplasm such as sarcoma with recommendations of further assessment with biopsy.

**Fig. 3:**
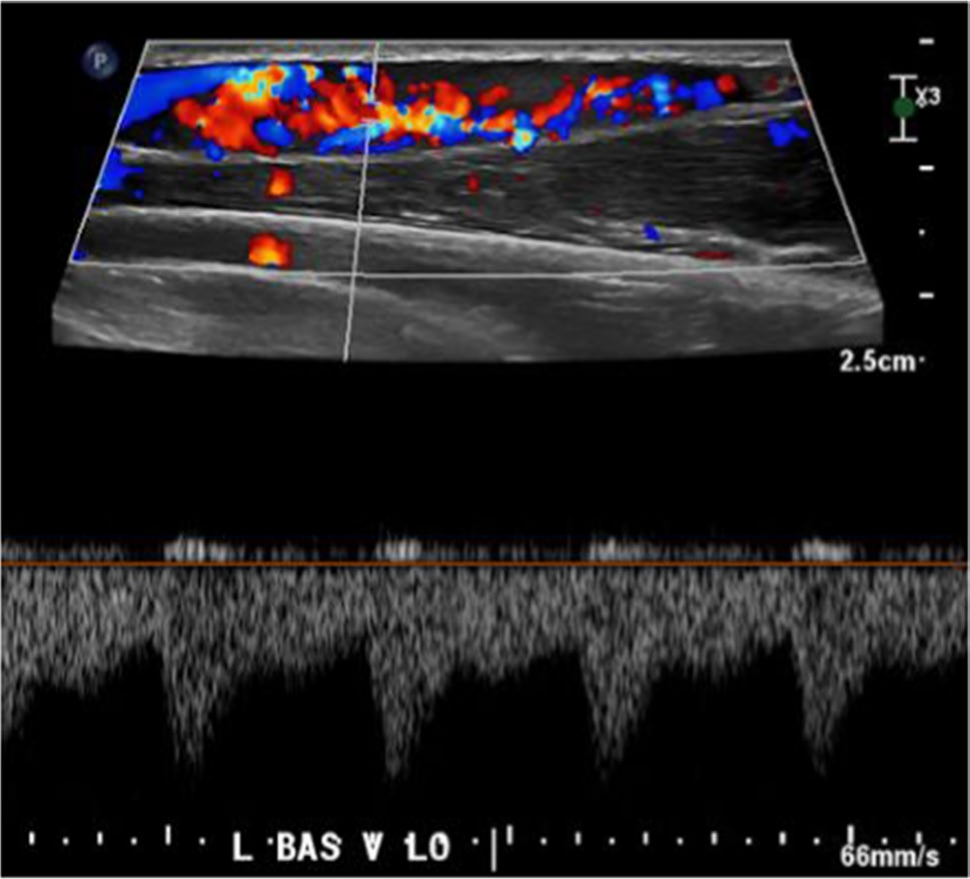
At the time of biopsy, there is increased vascularity with low resistance arterial waveform within the mass on longitudinal spectral Doppler ultrasound images at time of biopsy

Ultrasound-guided biopsy was preformed two-months following initial detection. At this time, the intravascular mass demonstrated increased size and vascularity (Fig. 3). Multiple 1–2 cm biopsies were obtained through the mass using 18- and 20-gauge needles, yielding several core specimens and tissue fragments.

**Fig. 4:**
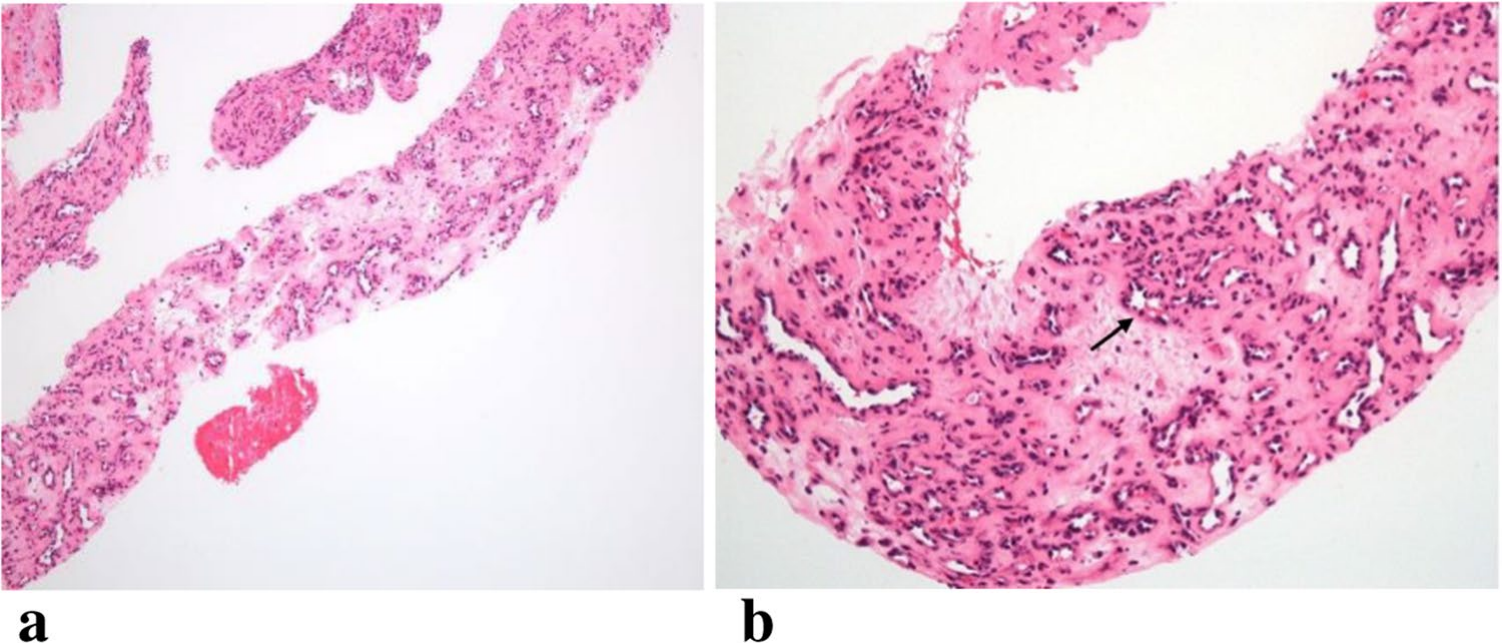
Histopathological appearance of the lesion. (**a**) At 10 × magnification, the specimen demonstrates small vessel proliferation in a fibromyxoid stroma with some variation in the degree of collagenization. (**b**) At 20 × magnification, vessels with prominent endothelium characteristic of immature blood vessel formation are visualized (arrow)

The core needle biopsy revealed reactive vascular proliferation with features of organizing thrombus. At low power, there was small vessel proliferation in a fibromyxoid stroma. At higher magnification, the vessels demonstrate prominent endothelium typical of immature blood vessel formation (Fig. 4). Notably, the papillary fronds characteristically seen in IPEH were not visualized. The patient was treated conservatively with no further intervention.

## Discussion

The most common intravascular mass is a bland superficial or deep vein thrombus that can be easily distinguished from primary neoplasms by absence of internal vascularity on imaging. In our patient, the inflammatory state of Miller Fisher syndrome combined with IVIG therapy —both associated with increased thrombotic risk — provided a plausible setting for thrombus formation [9]. Primary intravascular solid masses are uncommon by imaging, and particularly in the peripheral vessels [10]. Leiomyomas, leiomyosarcomas and angiosarcomas may arise from the endothelial and smooth muscle vessel wall in medium to large veins and arteries [10, 11]. Epithelioid hemangioendothelioma is a low-grade vascular tumor in medium to large veins that may share similar features of a deep vein thrombus and may have intralesional calcification [12]. Intravascular fasciitis is a rare benign fibroblastic intravascular tumor which may present as a hypoechoic intravascular mass with increased flow on ultrasound [13–15]. Metastasis may also occur intravascularly, whether seeded from a site upstream or direct invasion, as is more common in renal cell carcinoma and hepatocellular carcinoma.

Organizing thrombus is an important benign entity to include in the differential of intravascular masses. Organizing thrombus is most commonly a histopathologic diagnosis describing a normal process of vessel recanalization related to thrombus healing, including formation of fibrous tissue and neovascularization. If the organizing thrombus becomes encapsulated, it may prevent resorption and may lead to progressive expansion.

Intravascular papillary endothelial hyperplasia is an exuberant form of organizing thrombus, described as appearing on ultrasound as a well-defined hypoechoic intravascular mass with peripheral or septal vascularity, and on MRI as iso- to hypointense on T1, heterogenous hyperintense on T2, and showing peripheral or heterogeneous enhancement [4, 5, 16–18]. In contrast, there are few reports in the literature of the imaging appearance of organizing thrombus likely because of their small size. Ryu et. al described the sonographic appearance of a small collection of organizing thrombi and organizing hematomas, of which only one case demonstrated internal vascularity [19].

The inciting factor for development of reactive endothelial papillary proliferation, which defines IPEH, as opposed to a more bland organizing thrombus is unknown [12]. The pure form of IPEH is thought to arise within damaged or dilated vessels, although they can also arise within a vascular mass or even in a hematoma [4]. Definitive diagnosis requires analysis of histological findings with IPEH demonstrating the characteristic reactive papillary proliferation of endothelial cells with a fibrinous core. Histological findings may differ depending on the age of the lesion with late stage IPEH demonstrating fusion of papillae. Other important characteristics that support an IPEH diagnosis include absence of numerous or atypical mitotic figures, pleomorphism, extravascular extension, and tissue necrosis [21–23].

It is important for general pathologists to be aware of this entity, as it can be mistaken histologically for an angiosarcoma [21].

In this case, the patient’s initial thrombus symptoms resolved with anticoagulation, however, given that the subsequent lesion developed in the same location, it is postulated that the organizing thrombus expanded on a background of residual basilic vein thrombus or developed de novo in a chronically altered vessel. The relatively large size, marked internal vascularity, and imaging appearance qualify it as an exuberant form of organizing hematoma with early recanalization. The absence of papillary architecture histologically precludes the definitive diagnosis of IPEH. It is possible that the papillary architecture was simply not captured due to sampling error or possible atypical histologic evolution of an older lesion. Notably, a very similar case has been previously reported in the literature by Lysyy et al. of an IPEH in a superficial vein in the forearm in the absence of prior trauma or thrombus [24]. Exuberant organizing thrombus should be considered in the differential of enhancing endovascular masses of the extremity, as in this case.

The patient elected for conservative management and no further intervention was performed. IPEH may be treated with a simple marginal excision yielding good prognosis and only rare instances of recurrence [21, 25, 26].

In summary, as demonstrated in this case report organizing thrombus is a benign histologic process of thrombus resorption which may become apparent on imaging when the response is robust or exuberant. IPEH is a recognized form of exuberant organizing thrombus and is defined histologically by papillary architecture. This may present on imaging as an intravascular mass with increased T2 signal and internal vascularity often with a dominant vessel as in this case. The knowledge of this entity is important to expand on the differential diagnosis of an intravascular mass.

## Data Availability

All authors declare that they had full access to all of the data in this study and the authors take complete responsibility for the integrity of the data and the accuracy of the data analysis.
