# Correction to: Intravascular organizing thrombus in the forearm: a unique imaging presentation

**DOI:** 10.1007/s00256-026-05139-w

**Published:** 2026-01-22

**Authors:** Priyanka Mitta, Constantine Burgan, John Huffman, Bonnie Moore, Thomas Winokur, Rachel Bass

**Affiliations:** 1 School of Medicine, University of Alabama at Birmingham, 1620 University Boulevard, Birmingham, AL 35233 USA; 2 Department of Radiology, University of Alabama at Birmingham, 619 19th Street South, JTN 338, Birmingham, AL 35249 USA; 3 Department of Pathology, University of Alabama at Birmingham, 619 19th Street South, NP 3541, Birmingham, AL 35249 USA

**Correction to: Skeletal Radiology (2025)**

10.1007/s00256-025-05044-8

The correct order of authors should be:

Priyanka Mitta, Constantine Burgan, John Huffman, Bonnie Moore, Thomas Winokur and Rachel Bass

and not

Rachel Bass, Priyanka Mitta, Constantine Burgan, John Huffman, Bonnie Moore and Thomas Winokur

The original article is corrected.

